# Diminuição dos Níveis Séricos do Receptor Solúvel da Oncostatina M (sOSMR) e Glicoproteína 130 (sgp130) em Pacientes com Doença Arterial Coronariana

**DOI:** 10.36660/abc.20220326

**Published:** 2023-04-04

**Authors:** Vanessa Mylenna Florêncio de Carvalho, Priscilla Stela Santana de Oliveira, Amanda Pinheiro Barros de Albuquerque, Moacyr Jesus Barreto de Melo Rêgo, Michelle Melgarejo da Rosa, Dinaldo Cavalcanti de Oliveira, Michelly Cristiny Pereira, Maira Galdino da Rocha Pitta

**Affiliations:** 1 Universidade Federal de Pernambuco Recife PE Brasil Universidade Federal de Pernambuco, Recife, PE – Brasil

**Keywords:** Biomarcadores, Doença das Coronárias, Imunidade, Oncostatina M, Glicoproteínas

## Abstract

**Fundamento:**

A oncostatina M (OSM) é uma citocina pleiotrópica que, após lesão arterial, demonstra ser expressa rapidamente.

**Objetivos:**

Correlacionar os níveis séricos da OSM, do receptor solúvel de oncostatina M (sOSMR) e da fração solúvel de glicoproteína 130 (sgp130) em pacientes com doença arterial coronariana (DAC) a parâmetros clínicos.

**Métodos:**

Os níveis de sOSMR e sgp130 foram avaliados por ELISA, enquanto os de OSM foram avaliados por Western Blot, em pacientes com SCC (n=100), pacientes com SCA (n=70) e 64 voluntários do grupo de controle sem manifestações clínicas da doença. Valores de p <0,05 foram considerados estatisticamente significativos.

**Resultados:**

Pacientes com DAC exibiram níveis significativamente mais baixos de sOSMR e sgp130 e níveis mais altos de OSM em comparação ao grupo de controle (ambos p <0,0001). A análise clínica mostrou níveis mais baixos de sOSMR em homens ([OR] = 2,05, p = 0,026), jovens (OR = 1,68, p = 0,0272), hipertensos (OR = 2,19, p = 0,041), fumantes (OR = 2,19, p = 0,017), pacientes que não apresentavam dislipidemia (OR = 2,32, p = 0,013), pacientes com infarto agudo do miocárdio [IAM] (OR = 3,01, p = 0,001) e pacientes não tratados com estatina (OR = 1,95, p = 0,031), antiplaquetário (OR = 2,46, p = 0,005), inibidores dos canais de cálcio (OR = 3,15, p = 0,028) e antidiabéticos (OR = 2,97, p = 0,005). Os níveis de sOSMR também foram correlacionados a sexo, idade, hipertensão e uso de medicamentos na análise multivariada.

**Conclusões:**

Nossos dados sugerem que o aumento dos níveis séricos de OSM e a diminuição dos níveis de sOSMR e sGP130 em pacientes com injúria cardíaca podem desempenhar um papel importante no mecanismo fisiopatológico da doença. Além disso, níveis mais baixos de sOSMR foram associados a sexo, idade, hipertensão e uso de medicamentos.

## Introdução

Doenças cardiovasculares representam a principal causa de morte em todo o mundo. Em 2019, ocorreram 171.246 óbitos atribuídos à DAC no Brasil, sendo a principal causa de morte em quase todas as suas Unidades Federativas (UF), com exceção de duas.^1^

A *doença arterial coronariana* (DAC) ocorre em consequência do mecanismo de lesão arterial. Ela se baseia na aterosclerose, doença que afeta as artérias íntima e média associada a acúmulos focais de lipídios e fibras colágenas difusas, e é caracterizada por elementos de resposta inflamatória crônica. A síndrome coronariana crônica (SCC) é definida como a DAC com um processo crônico decorrente de mudanças no estilo de vida, podendo se manifestar como angina estável (onde o paciente apresenta sintomas), ou isquemia detectada por exames complementares (isquemia silenciosa), enquanto as síndromes coronarianas agudas (SCA) são caracterizada por uma redução repentina no fornecimento de sangue ao coração. A terapia farmacológica e a revascularização invasiva são métodos de tratamento em ambos os casos. As DAC podem ficar “estáveis” por um longo período, mas é possível ocorrer uma situação instável devido à ruptura ou erosão da placa com a transição para uma SCA a qualquer momento (ou seja, ambas são formas de uma doença com o mesmo mecanismo subjacente). Sabe-se que os linfócitos T CD4+ ativados desempenham papéis importantes na produção de citocinas, que podem levar à inflamação e danos vasculares.^2 , 3^

A oncostatina M (OSM) é conhecida como uma citocina pleiotrópica da família IL-6 produzida por células T ativadas, monócitos, células dendríticas, neutrófilos e macrófagos e, desempenha papéis fundamentais na inflamação, neuroproteção, metabolismo, sobrevivência celular e remodelação tecidual. Além dessa base, a atividade da OMS na aterosclerose coronária foi descoberta com resultados distintos. A literatura revelou que a OSM ativa o correceptor gp130, uma glicoproteína transdutora de sinal relacionada à via JAK/STAT que é responsável pela hipertrofia e pela regeneração das células cardíacas.^4 - 6^ Como alternativa, a função da OSM também pode apontar para a progressão da placa aterosclerótica.^7 , 8^

A sinalização de OSM envolve a ligação da subunidade gp130 do receptor do fator inibidor de leucemia (LIFR) [LIFRβ/gp130] e à subunidade gp130 e receptor da OSM [OSMRβ/gp130].^9 - 11^ A fração solúvel de OSMR (sOSMR) é formada por uma variedade de mecanismos, incluindo clivagens proteolíticas dos domínios extracelulares do receptor, a fragmentação de um resíduo de glicosilfosfatidilinositol e o splicing alternativo de transcritos de RNA.^12 - 14^

A sgp130, uma antagonista natural de IL-6, é alternativamente processada a partir do mRNA ou eliminada do ectodomínio de gp130 ligado à membrana. Tem propriedades anti-inflamatórias principalmente por meio da inibição endógena da transinalização da IL-6.^15 - 17^

Estudos pré-clínicos usando a proteína sgp130 mostraram efeitos terapêuticos razoáveis em modelos animais de artrite reumatoide, lúpus eritematoso, e doença inflamatória intestinal.^18 , 19^ No entanto, o papel da sgp130 nas DAC permanece obscuro. Poucos estudos avaliaram a associação entre os níveis séricos de OSM e a gravidade da DAC.^20^ Assim, em nosso estudo, avaliamos os níveis séricos de OSM solúvel, OSMR e sgp130 em pacientes com DAC. Além disso, investigamos como a expressão sérica de OSM, OSMR e sgp130 se correlaciona às variáveis clínicas dos pacientes.

## Métodos

### População do estudo

A população deste estudo foi composto por pacientes adultos (maiores de 18 anos), com diagnóstico clínico de SCC (70% ou mais de obstrução da luz vascular vista por CATE), pacientes com diagnóstico de SCA (presença de trombo oclusivo na luz vascular vista por CATE) e sujeitos do grupo de controle. O sangue dos pacientes com SCA foi coletado até no máximo 3 dias após a internação, pois as coletas foram realizadas às segundas, quartas e sextas-feiras. O critério utilizado para diferenciar pacientes com SCA entre infarto agudo do miocárdio - IAM - (STEMI vs. NSTEMI) e angina instável foi a angiocoronariografia (ACG) realizada pelo cardiologista, além do eletrocardiograma e marcadores bioquímicos de necrose miocárdica como CK-MB e todas as frações de troponina.

Os acompanhantes dos pacientes que se voluntariaram e atenderam aos critérios de inclusão (idade > 18 anos e sem manifestações clínicas da DAC) formaram o grupo controle. As características da linha de base são apresentadas na Tabela 1 .

**Tabela 1:** – Características clínicas de pacientes com síndrome coronariana crônica (SCC), síndrome coronariana aguda (SCA) e grupo de controle

Características	SCC (N = 100)	SCA (N = 70)	Controle (N = 64)
Idade (anos)	63,32 ± 9,8	63,4 ± 11,9	58,97± 11,2
Sexo (masculino/feminino)	59/41	49/21	40/24
**Fatores de risco**			
Hipertensão, n (%)	78 (78%)	55 (78,57%)	36 (56,25%)
Diabetes, n (%)	43 (43%)	31 (44,28%)	-
Dislipidemia, n (%)	30 (30%)	23 (33,85%)	17 (26,56%)
Doença cardiovascular, n (%)	12 (12%)	6 (8,57%)	-
Acidente vascular cerebral, n (%)	6 (6%)	3 (4,28%)	-
Revascularização, n (%)	12 (12%)	6 (8,57%)	-
*IAM, n (%)	-	29 (41,42%)	-
Stent, n (%)	18 (18%)	14 (20%)	-
Tabagismo, n (%)	39 (39%)	31 (44,28%)	5 (7,81%)
**Medicamentos**			
Betabloqueador, n (%)	52 (52%)	36 (51,42%)	6 (9,38%)
BCC, n (%)	11 (11%)	8 (11,42%)	7 (10,94%)
IECA/BRA, n (%)	38 (38%)	23 (32,85%)	9 (14,06%)
ARA, n (%)	26 (26%)	10 (14,28%)	19 (29,69%)
Diurético, n (%)	21 (21%)	11 (15,71%)	14 (21,86%)
Antidiabético, n (%)	22 (22%)	15 (21,42%)	
Estatina, n(%)	60 (60%)	32 (45,71%)	-
Insulina, n (%)	3 (3%)	3 (4,28%)	8 (12,5%)
Nitratos, n (%)	15 (15%)	4 (5,71%)	-
Agente antiplaquetário, n (%)	67 (67%)	35 (50%)	-
Fibratos, n (%)	3 (3%)	1 (1,42%)	-
Anti-isquêmicos, n (%)	5 (5%)	-	-

*IAM: Infarto agudo do miocárdio - [IAM foi o motivo da hospitalização]. †BCC: bloqueador de canais de cálcio; IECA/BRA: enzima conversora da angiotensina; ARA: antagonista do receptor da angiotensina.

Excluído da análise foram os pacientes com doença hepática grave, doença renal crônica de estágio IV ou V, discrasia sanguínea, câncer ativo, metástase ativa, aqueles em quimioterapia ou radioterapia, pacientes com expectativa de vida <1 ano e pacientes em uso de imunossupressores.

Para todos os grupos, foi realizada a amostragem por conveniência. No total, foram coletadas 170 amostras de sangue dos pacientes. Os pacientes foram divididos em dois subgrupos: pacientes com síndrome coronariana crônica (SCC; n=100) e pacientes com síndrome coronariana aguda (SCA; n=70 – sendo n=29 pacientes SCA com IAM, e n=41 pacientes SCA com angina), junto com o grupo controle (n=64).

O protocolo do estudo foi aprovado pelo comitê de ética em pesquisa da Universidade Federal de Pernambuco (CAAE: 16356619.7.0000.5208).

### Definição das variáveis estudadas

As variáveis clínicas de interesse foram coletadas por meio da aplicação de questionários. Todas as definições de comorbidades foram feitas com base no autorrelato dos pacientes.

A hipertensão foi diagnosticada com base no uso de anti-hipertensivos ou nas medidas de pressão arterial sistólica/diastólica ≥140/90 mmHg. Diabetes mellitus foi definido como uso de insulina ou hipoglicemiantes orais, ou glicemia de jejum ≥126 mg/dL. A hiperlipidemia foi diagnosticada com base na concentração de colesterol total em jejum ≥200 mg/dL, concentração de triglicerídeos ≥150 mg/dL, ou uso de hipolipemiantes. Consideraram-se portadores de doença cardiovascular aqueles que apresentavam arritmia, angina, cardiomiopatia, insuficiência cardíaca congestiva ou já haviam sofrido acidente vascular cerebral (AVC).

### Ensaio de imunoabsorção enzimática (ELISA)

Amostras de sangue foram coletadas antes da angiografia coronária. Foram obtidas amostras de sangue venoso periférico de todos os sujeitos em tubos, sem anticoagulantes. Em sequência, o soro foi separado por centrifugação e armazenado a -80 °C até o uso. Os níveis séricos de sOSMR e sgp130 foram medidos em pacientes com DAC e controlados por ELISA, usando kits específicos (R&D Systems, Minneapolis, EUA e eBioscience, San Diego, CA), conforme o protocolo do fabricante. O limite de detecção mais baixo do ensaio foi de 156,25 pg/mL para a sOSMR e 78,125 pg/mL para a sgp130. Não foram detectados níveis séricos de OSM pela metodologia citada acima. O limite de detecção mais baixo do ensaio foi de 15,2 pg/mL. Portanto, os níveis séricos de OSM foram medidos por Western Blot.

### Medição de OSM

A expressão proteica da OSM no soro dos pacientes foi realizada por Western Blot.^21^ Uma alíquota de soro de cada paciente foi diluída 1:10 em água MiliQ e a quantificação da proteína foi determinada pelo kit BCA Protein Assay conforme as instruções do fabricante (Sigma-Aldrich®). Após a análise de absorbância, 50μg de proteínas foram submetidos à eletroforese em gel de poliacrilamida 10% contendo SDS (SDS-PAGE) e transferidos para membrana de nitrocelulose (GE Healthcare Life Sciences). O bloqueio de locais não específicos foi realizado incubando a membrana com TBST-BSA 5% a 4°C durante a noite. As membranas foram incubadas com o anticorpo primário monoclonal oncostatina M (OSM) de coelho (ColorBurst®) diluído a 1:1000 em TBST-BSA 5% às 4 horas TA. Em seguida, as membranas foram incubadas com os respectivos anticorpos secundários conjugados com HRP (1:5000). Proteínas imunomarcadas conjugadas com HRP foram detectadas pelo método de quimioluminescência aprimorada (ECL, GE Healthcare Life Sciences).

### Análise estatística

Os dados foram analisados usando GraphPad Prism (versão 6.0, San Diego, CA). A normalidade das amostras foi verificada com o teste de D’Agostino, e as variáveis contínuas foram expressas como média ± desvio padrão (DP), se normalmente distribuídas, ou como medianas [faixa interquartil [FIQ] (25º-75º percentil)], se não apresentarem distribuição gaussiana. A mediana também foi o critério utilizado para categorizar os grupos em maiores e menores níveis séricos de sOSMR e sgp130. Para análise de citocinas foi utilizado o teste não paramétrico de Kruskal-Wallis, seguido do post hoc de Brown-Forsythe, assim como a correlação de Spearman para variáveis contínuas; o teste qui-quadrado e exato de Fisher foi usado para variáveis categóricas. As variáveis categóricas foram descritas por frequências absolutas e relativas.

Além disso, a análise de variância de uma via (ANOVA), seguida pelos testes post hoc de Brown-Forsythe e Bartlett, foram usados para avaliar a expressão proteica da OSM por Western Blot. A análise multivariada também foi realizada para discernir quais parâmetros tinham valor preditivo independente em relação aos níveis séricos de sOSMR, analisados de maneira inferencial, usando o teste qui-quadrado de Pearson ou teste exato de Fisher. O modelo de análise de regressão utilizado foi o logístico. Valores de p <0,05 foram considerados estatisticamente significativos.

## Resultados

### Níveis séricos de sOSMR e sgp130

**Figura 1:**
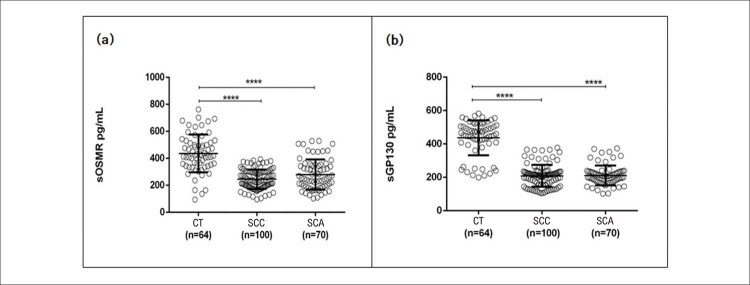
– Distribuição geral dos níveis séricos de sOSMR e sgp130 da população do estudo. A) Níveis séricos de sOSMR (receptor solúvel da oncostatina M) em pg/mL nos sujeitos do grupo de controle (CT) e nos grupos síndrome coronariana crônica (SCC) e síndrome coronariana aguda (SCA). B) Níveis séricos de sgp130 (glicoproteína 130, fração solúvel) em pg/mL nos sujeitos do grupo de controle (CT) e nos grupos síndrome coronariana crônica (SCC) e síndrome coronariana aguda (SCA). ****p <0.0001 vs. controle: Significativo depois análise do teste de Kruskal-Wallis.

As manifestações clínicas dos participantes estão resumidas na Tabela 1 . Pacientes com SCC e SCA apresentaram níveis séricos significativamente mais baixos de sOSMR em comparação com o grupo controle, conforme mostrado na Figura 1a . Além disso, foi detectada menor expressão sérica de sgp130 na SCC e na SCA quando comparada ao grupo de controle, conforme mostrado na Figura 1b . Nenhuma diferença significativa foi detectada nos níveis séricos de sOSMR e sgp130 entre pacientes com SCC quando comparados aos de SCA.

### Níveis séricos de sOSMR de acordo com sexo e idade

**Figura 2:**
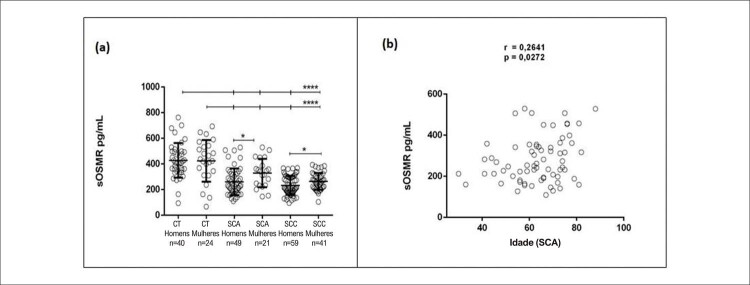
– Distribuição geral dos níveis séricos de sOSMR por sexo e idade. A) Distribuição dos nos grupos controles (CT) e nos grupos síndrome coronariana crônica (SCC) e síndrome coronariana aguda (SCA) entre homens e mulheres níveis séricos de pg/mL (receptor solúvel da oncostatina M) sOSMR. B) Correlação entre os níveis de sOSMR (receptor solúvel da oncostatina M) em pg/mL e a idade de pacientes com SCA. ****p <0,0001 vs. grupo de controle: Significativo depois análise do teste de Kruskal-Wallis; * p <0,05 após a correlação de Spearman.

A Figura 2a revela que pacientes do sexo masculino com SCA e SCC apresentaram níveis séricos de sOSMR mais baixos do que mulheres com as mesmas condições. Além disso, níveis séricos mais altos de sOSMR foram detectados em mulheres com SCA em comparação com SCC.

Entre os pacientes com SCA, a idade apresentou uma correlação positiva significativa com os níveis séricos de sOSMR, como mostrado na Fig. 2b . A análise de correlação também foi realizada nos grupos de SCC e de controle, mas não houve resultados significativos para nenhum dos grupos [dados não mostrados].

### Níveis séricos de OSM por Western Blot

**Figura 3:**
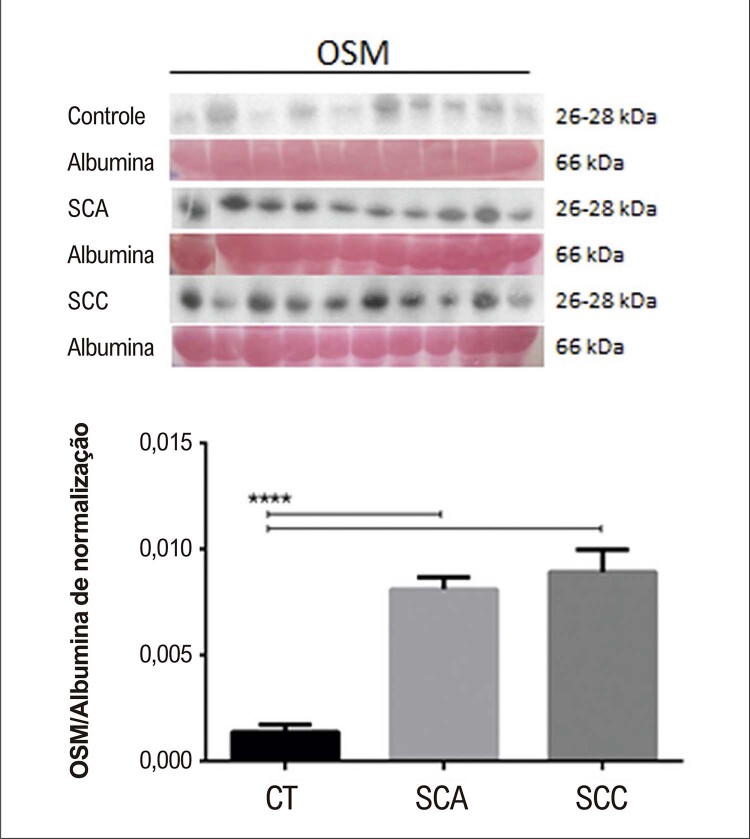
– Expressão proteica de oncostatina M (OSM) entre indivíduos controle (CT), pacientes com síndrome coronariana aguda (SCA) e pacientes com síndrome coronariana crônica (SCC) após normalização com albumina corada por Ponceau S. **** p <0,0001 vs. o grupo de controle: Significativo depois análise de variância (ANOVA).

O soro de 10 pacientes em cada grupo foi selecionado de acordo com os critérios de representatividade da amostra (idade média de 60,5, 65,2 e 63,9 anos nos grupos CT, SCA e SCC, respectivamente). A Figura 3 indica expressão aumentada de OSM em grupos de SCA e de SCC em comparação com o grupo controle. Não houve diferença significativa nos níveis séricos de OSM entre os grupos de SCA e de SCC. A normalização para proteína constitucional foi realizada com albumina, corada pela técnica de Ponceau S 0,01%,^22^ pois a amostra utilizada para o Western Blot foi sangue total.

### Níveis séricos e variáveis clínicas

Os resultados indicam uma associação significativa entre os níveis séricos de sOSMR e pacientes com hipertensão, dislipidemia, acidente vascular cerebral, revascularização, infarto agudo do miocárdio (IAM) e pacientes tabagistas ( Tabela 1 do Material Suplementar ).

Observando os medicamentos, o uso de estatinas, agentes antiplaquetários, insulina, bloqueadores dos canais de cálcio (BCC) e medicamentos antidiabéticos está positivamente associado aos níveis séricos de sOSMR ( Tabela 2 do Material Suplementar ).

**Tabela 2:** – Resultados da regressão logística para a porcentagem de pacientes com níveis baixos de sOSMR segundo variáveis independentes, com p <0,20 para serem incluídos na análise e p <0,20 para permanecer no modelo

Variável	Bivariada		Padronizado	
	
	OR e IC de 95.0%	p-valor	OR e IC de 95.0%	p-valor
**Sexo**		0,026*		
Homens	2,05 (1,09 a 3,89)		1,82 (0,89 a 3,75)	0,102
Mulheres	1,00		1,00	
**Idade**		0,091		
Até 60 anos	1,68 (0,92 a 3,09)		2,28 (1,12 a 4,65)	0,023*
Acima de 60 anos	1,00		1,00	
**Hipertensão**		0,041*		
Sim	2,19 (1,02 a 4,64)		2,69 (1,15 a 6,30)	0,023*
Não	1,00		1,00	
**Dislipidemia**		0,013*		
Sim	1,00		1,00	
Não	2,32 (1,86 a 4,52)		2,56 (1,19 a 5,51)	0,016*
**Infarto do miocárdio agudo**		0,001*		
Sim	1,00		1,00	
Não	3,01 (1,54 a 5,90)		2,99 (1,41 a 6,32)	0,004*
**Agente antiplaquetário**		0,005*		
Sim	1,00		1,00	
Não	2,46 (1,31 a 4,62)		2,56 (1,25 a 5,25)	0,010*
**Bloqueador dos canais de cálcio**		0,028*		
Sim	1,00		1,00	
Não	3,15 (1,08 a 9,19)		4,13 (1,26 a 13,54)	0,019*

* valores de significância (valores de p <0,05).

A Tabela 2 mostra que os níveis séricos circulantes de sOSMR foram independentemente associados com sexo, idade, hipertensão, ausência de histórico de dislipidemia e infarto agudo do miocárdio, e não uso de agentes antiplaquetários e BBC.

## Discussão

O presente estudo demonstrou não apenas expressão sérica diminuída de sOSMR, mas também concentrações aumentadas de OSM sérico em pacientes com SCC ou SCA.

A OSM aparece no coração após dano cardíaco, a fim de promover a sobrevivência celular e o reparo tecidual.^23^ No estudo de Hu et al. (2017), a OSM atenuou a remodelação do ventrículo esquerdo e restaurou a densidade das cristas mitocondriais.^6^ Uma forma solúvel fragmentada do receptor OSM foi identificada (ou seja, sOSMR), responsável por uma atividade antagonista no de receptor OSM.^16 , 17^

Além disso, detectamos a expressão sérica da OSM em pacientes com DAC por meio da análise de Western Blot. A ausência de detecção via ELISA pode assemelhar-se à diferença no reconhecimento do epítopo do anticorpo usado neste estudo ou os níveis séricos da OSM podem estar abaixo do limite de detecção pelo ELISA. É importante notar que vários outros fatores, incluindo a presença de receptores solúveis e receptores antagonistas, também são conhecidos por influenciar a quantificação de citocinas no soro.^24^

Por um lado, a literatura sugere que a OSM é uma citocina rara (ou seja, não comumente expressa) em que seus níveis elevados no soro indicam proteção e reparo tecidual após doenças cardíacas.^23^ Por outro lado, o estudo de Ikeda et al. (2021) sugeriu uma associação positiva entre os níveis séricos de OSM e o desenvolvimento de estenose.

A ligação da OSM na sOSMR requer a ativação da gp130 e resulta na inibição da atividade da OSM, sinalização envolvida no reparo tecidual após uma lesão cardíaca.^11^ Em nosso estudo, níveis elevados de OSM foram observados em pacientes com lesão cardíaca quando comparados ao grupo de controle, enquanto níveis reduzidos de sOSMR e sGP130 foram observados no mesmo grupo de pacientes. O grupo controle não apresentou nenhuma lesão cardíaca ou dano tecidual que desencadeasse mecanismos endógenos de reparo, como o aumento da expressão de OSM observado nos grupos de SCA e SCC. Assim, nossos resultados sugerem que a expressão aumentada de OSM em pacientes com SCA e SCC pode indicar um papel importante no mecanismo fisiopatológico da lesão cardíaca. No entanto, devem ser realizados mais estudos sobre o acompanhamento da evolução da doença e expressão sérica de OSM/sOSMR/sGP130 para esclarecer a hipótese de dano ou reparo tecidual.

Apesar dos dados de que a OSM pode promover a expressão de OR,^12^ poucas tentativas foram feitas para explorar tal sinalização.^25^ Uma hipótese prepara o caminho para a ligação de sOSMR na OSM (ou seja, OSMRβ) que pode interromper a ativação da OSM. Bloquear a sinalização OSM requer a ação da sgp130. De fato, no presente estudo, observamos que pacientes com DAC também apresentam baixos níveis de sgp130 quando comparados ao grupo controle.

No entanto, na literatura, a associação entre os níveis de sgp130 e doenças cardiovasculares é controversa. Um estudo de uma população idosa com insuficiência cardíaca mostrou que níveis aumentados de sGP130 estão relacionados à mortalidade cardiovascular.^26^ Além disso, altos níveis de sGP130 predizem mau prognóstico em pacientes com histórico de infarto do miocárdio.^27^ Por outro lado, uma pesquisa de caso-controle baseada em uma população muito maior propôs que altos níveis de sGP130 têm efeitos protetores contra a ocorrência de infarto do miocárdio.^28^ Um estudo sorológico indicou que ambos os pacientes com DAC em condição instável apresentam níveis significativamente reduzidos de sgp130 endógeno. Além disso, um estudo recente demonstrou que os níveis de sgp130 eram significativamente mais baixos em pacientes com DAC instável ou progressiva.^29^ Ademais, o estudo de Zhou et al. descobriu que o nível de sGP130 de 136,01 ng/mL foi um ponto de corte eficaz para prever as DAC.^30^ Da mesma forma, também sugerimos que os níveis de sgp130 podem ser biomarcadores úteis para a identificação de DAC. Foi observada uma correlação positiva entre os níveis séricos de OSM e a presença de pacientes com DAC, corroborando o estudo de Ikeda et al. (2021). Altos níveis de OSM entre pacientes com DAC podem aumentar os mecanismos compensatórios para a sobrevivência celular.^31^ No estudo de Wahl et al., a OSM equilibra as respostas inflamatórias, suprimindo a inflamação, em modelos murinos de doenças inflamatórias crônicas, incluindo artrite reumatoide e esclerose múltipla.^32^ Nossos resultados também indicam que a idade se correlaciona aos níveis séricos de sOSMR. O estudo de Hartel et al. (2005) mostrou o contrário. Eles observaram que os níveis de citocinas como TNF, INF-γ e IL-2 aumentam progressivamente com a idade.^33^ Nossos estudos sugeriram que quanto menor a expressão de OSM em pacientes idosos, menor a probabilidade de desenvolvimento de DAC. Portanto, a OSM pode aumentar a proteção nessa população. Ao contrário, a literatura tem revelado que a gravidade da DAC aumenta com a idade, o que tem sido atribuído à maior prevalência de obstrução física das artérias coronárias causada pela aterosclerose.^34 , 35^

Em relação ao sexo dos pacientes, os resultados sugerem que os homens apresentam níveis séricos de sOSMR mais baixos do que as mulheres. Estudos anteriores mostraram que a presença de estrogênio durante o período fértil prolonga o início da doença aterosclerótica em mulheres.^36 , 37^ Um estudo clínico da Women’s Ischemia Syndrome Evaluation em 2003 revelou que mulheres jovens com deficiência endógena de estrogênio têm um risco sete vezes maior de desenvolver aterosclerose.^38^ Na ausência do benefício cardioprotetor do estrogênio, os homens presumivelmente requerem a ativação da via OSM em compensação.

Nosso estudo teve algumas limitações, incluindo um pequeno número de amostras e a heterogeneidade do tipo de medicamento usado pelos participantes. Além disso, seria interessante associar sOSMR e sgp130 a marcadores clássicos de dano tecidual e metabolismo lipídico envolvidos na angiocoronariografia. Para resumir, é apresentada uma Figura central com os principais resultados deste artigo, para esclarecer ideias.

**Figura Central:**
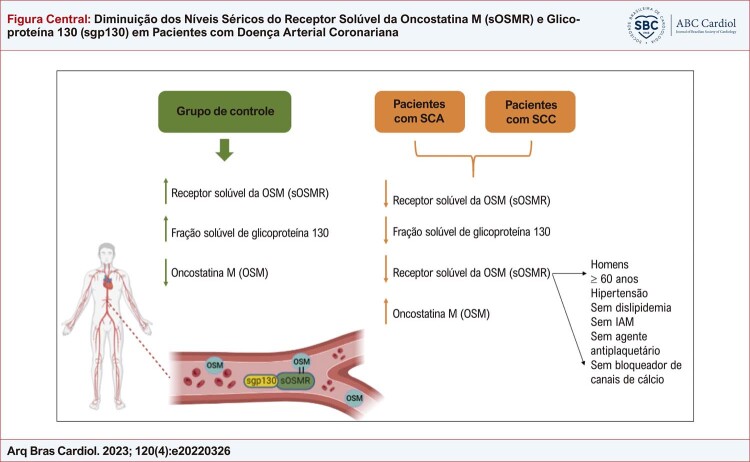
: Diminuição dos Níveis Séricos do Receptor Solúvel da Oncostatina M (sOSMR) e Glicoproteína 130 (sgp130) em Pacientes com Doença Arterial Coronariana

Figura central sintetizando os principais resultados desta pesquisa.

## Conclusões

Nossos dados sugerem que o aumento dos níveis séricos de OSM e a diminuição dos níveis de sOSMR e sGP130 em pacientes com injúria cardíaca podem desempenhar um papel importante no mecanismo fisiopatológico da doença. Além disso, níveis mais baixos de sOSMR foram associados a sexo, idade, hipertensão e uso de medicamentos. Devem ser conduzidos estudos adicionais sobre o acompanhamento do desfecho da doença e expressão sérica de OSM/sOSMR/sGP130 com maior número de pacientes, terapia controlada e associação com biomarcadores de dano ou reparo tecidual para fortalecer nossa hipótese.

## * Material suplementar

Para informação adicional, por favor, clique aqui .
