# Supplementary material for: Diminuição dos Níveis Séricos do Receptor Solúvel da Oncostatina M (sOSMR) e Glicoproteína 130 (sgp130) em Pacientes com Doença Arterial Coronariana
Source: Arq Bras Cardiol. 2023 Apr 4;120(4):e20220326. [Article in Portuguese] doi: 10.36660/abc.20220326 (PMC10263435; doi:10.36660/abc.20220326)
Supplement: Translation

# Translation: Decreased Serum Levels of Soluble Oncostatin M Receptor (sOSMR) and Glycoprotein 130 (sgp130) in Patients with Coronary Artery Disease

**Authors:** Vanessa Mylenna Florêncio de Carvalho, Priscilla Stela Santana de Oliveira, Amanda Pinheiro Barros de Albuquerque, Moacyr Jesus Barreto de Melo Rêgo, Michelle Melgarejo da Rosa, Dinaldo Cavalcanti de Oliveira, Michelly Cristiny Pereira, Maira Galdino da Rocha Pitta

**Affiliations:** 1 Brazil Universidade Federal de Pernambuco, Recife, PE – Brazil

**Keywords:** Biomarkers, Coronary Disease, Immunity, Oncostatin M, Glycoproteins

## Abstract

**Background:**

Oncostatin M (OSM) is a pleiotropic cytokine which, after arterial injury, has proven to be to be rapidly expressed.

**Objectives:**

To correlate the serum levels of OSM, soluble OSM receptor (sOSMR), and soluble fraction of glycoprotein 130 (sgp130) in patients with coronary artery disease (CAD) with clinical parameters.

**Methods:**

Levels of sOSMR and sgp130 were evaluated by ELISA and OSM by Western Blot, in patients with CCS (n=100), patients with ACS (n=70), and 64 control volunteers without clinical manifestations of the disease. P-values < 0.05 were considered to be statistically significant.

**Results:**

CAD patients exhibited significantly lower levels of sOSMR and sgp130 and higher levels of OSM when compared to the controls (both p < 0.0001). Clinical analysis displayed, lower levels of sOSMR in men ([OR] = 2.05, p = 0.026), youth (OR = 1.68, p = 0.0272), hypertensives (OR = 2.19, p = 0.041), smokers (OR = 2.19, p = 0.017), patients that did not present dyslipidemia (OR = 2.32, p = 0.013), patients with Acute Myocardial Infarction [AMI] (OR = 3.01, p = 0.001) and patients not treated with statin (OR = 1.95, p = 0.031), antiplatelet agent (OR = 2.46, p = 0.005), inhibitors of calcium channels (OR = 3.15, p = 0.028), and antidiabetic drugs (OR = 2.97, p = 0.005). The levels of sOSMR were also correlated with gender, age, hypertension, and use of medications in multivariate analysis.

**Conclusions:**

Our data suggest that the enhanced serum levels of OSM, and decreased levels of sOSMR and sGP130 in patients with cardiac injury may play an important role in the pathophysiological mechanism of the disease. Furthermore, lower levels of sOSMR were associated with gender, age, hypertension, and the use of medications.

## Introduction

Cardiovascular diseases represent the leading cause of death worldwide. In 2019, there were 171,246 deaths attributed to CAD in Brazil, which was the main cause of death in almost all of its states, with the exception of two.^1^

Coronary artery disease (CAD) occurs as a consequence of the arterial injury mechanism. It is based on atherosclerosis, a disease that affects the intima and medium arteries, associated with focal accumulations of lipids and diffuse collagen fibers, and is characterized by elements of a chronic inflammatory response. Chronic coronary syndrome (CCS) defines CAD as a chronic process that results from lifestyle changes, which may appear as stable angina (where the patient has symptoms), or ischemia detected by complementary exams (silent ischemia), whereas acute coronary syndromes (ACS) are characterized by a sudden reduction in blood supply to the heart. Pharmacological therapy and invasive revascularization are methods of treatment in both cases. CAD can be “stable” for a long period of time, but an unstable situation due to plaque rupture or erosion with the transition to an ACS is possible at any time, (i.e., both are forms of a disease with the same underlying mechanism). It is known that activated CD4 + T lymphocytes play important roles in the production of cytokines, which can lead to inflammation and vascular damage.^2 , 3^

Oncostatin M (OSM) is well-known as a pleiotropic cytokine in the IL-6 family produced by activated T cells, monocytes, dendritic cells, neutrophils, and macrophages, and it plays fundamental roles in inflammation, neuroprotection, metabolism, cell survival, and tissue remodeling. Moreover, OMS’s activity in coronary atherosclerosis has been uncovered with distinguished outcomes. The literature has unveiled that OSM activates the gp130 co-receptor, a signal transducer glycoprotein related to the JAK / STAT pathway, which is responsible for cardiac cell hypertrophy and regeneration.^4 - 6^Alternatively, the function of OSM might also point toward the progression of atherosclerotic plaque.^7 , 8^

OSM signaling involves the binding of the gp130 subunit of leukemia inhibiting factor receptor (LIFR) [LIFRβ / gp130] and to the gp130 subunit and OSM’s receptor [OSMRβ / gp130].^9 - 11^The soluble fraction of OSMR (sOSMR) is formed via a variety of mechanisms, including proteolytic cleavages of the receptor’s extracellular domains, the fragmentation of a glycosylphosphatidylinositol residue, and the alternative splicing of RNA transcripts.^12 - 14^

Sgp130, a natural IL-6 antagonist, is alternatively processed from the mRNA or eliminated from the membrane-bound gp130 ectodomain. It contains anti-inflammatory properties, mainly through endogenous inhibition of IL-6 trans-signaling.^15 - 17^

Pre-clinical studies using sgp130 proteins have shown reasonable therapeutic effects in animal models of rheumatoid arthritis, lupus erythematosus, and inflammatory bowel disease.^18 , 19^However, the role of sgp130 in CAD remains obscure. Few studies have evaluated the association between serum levels of OSM and the severity of CAD.^20^Thus, in our study, we evaluated the serum levels of soluble OSM, OSMR, and sgp130 in patients with CAD. Additionally, we investigated how OSM, OSMR, and sgp130 serum expression correlate with the clinical variables of patients.

## Methods

### Study population

The population of this study consisted of adult patients (aged > 18 years), with a clinical diagnosis of CCS (70% or more of vascular lumen obstruction seen through CATE), patients diagnosed with ACS (presence of occlusive thrombus in the vascular lumen seen through the CATE), and controls. Blood from patients with ACS was collected up to a maximum of three days after hospital admission, since collections were performed on Mondays, Wednesdays, and Fridays. The criterion used to differentiate patients with ACS between Acute Myocardial Infarction -AMI- (STEMI *vs* NSTEMI) and unstable angina was the coronary angiography (CAG) performed by the cardiologist, in addition to the electrocardiogram and biochemical markers of myocardial necrosis such as CK-MB and all troponin fractions. The companions of patients who volunteered and met the inclusion criteria (aged > 18 years and without clinical manifestations of the CAD) formed the control group. Detailed clinical characteristics are found in Table 1 .

**Table 1:** – Clinical characteristics of patients with Chronic Coronary Syndrome (CCS), Acute Coronary Syndrome (ACS), and Control Group

Characterístics	CCS (N = 100)	ACS (N = 70)	Control (N = 64)
Age (years)	63.32 ± 9.8	63.4 ± 11.9	58.97± 11.2
Sex (male/female)	59/41	49/21	40/24
**Risk factors**			
Hypertension, n (%)	78 (78%)	55 (78.57%)	36 (56.25%)
Diabetes, n (%)	43 (43%)	31 (44.28%)	-
Dyslipidemia, n (%)	30 (30%)	23 (33.85%)	17 (26.56%)
Cardiovascular disease, n (%)	12 (12%)	6 (8.57%)	-
Stroke, n (%)	6 (6%)	3 (4.28%)	-
Revascularization, n (%)	12 (12%)	6 (8.57%)	-
*AMI, n (%)	-	29 (41.42%)	-
Stent, n (%)	18 (18%)	14 (20%)	-
Smoking, n (%)	39 (39%)	31 (44.28%)	5 (7.81%)
**Medications**			
Beta blocker, n (%)	52 (52%)	36 (51.42%)	6 (9.38%)
BCC, n (%)	11 (11%)	8 (11.42%)	7 (10.94%)
ACEI/BRA, n (%)	38 (38%)	23 (32.85%)	9 (14.06%)
ARA, n (%)	26 (26%)	10 (14.28%)	19 (29.69%)
Diuretic, n (%)	21 (21%)	11 (15.71%)	14 (21.86%)
Antidiabetic, n (%)	22 (22%)	15 (21.42%)	
Statin, n (%)	60 (60%)	32 (45.71%)	-
Insulin, n (%)	3 (3%)	3 (4.28%)	8 (12.5%)
Nitrates, n (%)	15 (15%)	4 (5.71%)	-
Antiplatelet agent, n (%)	67 (67%)	35 (50%)	-
Fibrates, n (%)	3 (3%)	1 (1.42%)	-
Anti-ischemic, n (%)	5 (5%)	-	-

*AMI: acute myocardial infarction - [AMI was the reason for hospitalization]. †BCC: Blocker Calcium Channels; ACEI/BRA: angiotensin-converting enzyme; ARA: angiotensin receptor antagonist.

Excluded from the analysis were patients with severe liver disease, chronic kidney disease stages IV or V, blood dyscrasia, active cancer, active metastasis, those in chemotherapy or radiotherapy, patients with a life expectancy <1 year, and patients using immunosuppressants.

For all groups, sampling was performed for convenience. In total, 170 blood samples of patients were collected. The patients were divided into two subgroups: patients with Chronic Coronary Syndrome (CCS; n=100) and patients with Acute Coronary Syndrome (ACS; n=70 – being n=29 patients ACS with AMI, and n=41 patients ACS with unstable angina), along with the control group (n=64).

The study protocol was approved by the research ethics committee of Universidade Federal de Pernambuco (CAAE: 16356619.7.0000.5208).

### Definition of the studied variables

The clinical variables of interest were collected through the application of questionnaires. All definitions of comorbidities were made based on the patients’ self-report.

Hypertension was diagnosed based on the use of antihypertensive drugs or the measurements of systolic/diastolic blood pressure ≥140/90 mmHg. Diabetes mellitus was defined as the use of insulin or oral hypoglycemic drugs, or a fasting glucose level ≥126 mg/dL. Hyperlipidemia was diagnosed based on fasting total cholesterol concentration ≥200 mg/dL, triglyceride concentration ≥150 mg/dL, or the use of lipid-lowering agents. Cardiovascular disease was considered to be those who had arrhythmia, angina, cardiomyopathy, congestive heart failure, or who had already had a stroke.

### Enzyme-linked immunosorbent assay (ELISA)

Blood samples were taken before coronary angiography. Peripheral venous blood samples were obtained from all subjects in tubes without anticoagulants. Subsequently, the serum was separated by centrifugation and stored at -80°C until use. Serum levels of sOSMR and sgp130 were measured in CAD patients and controlled by ELISA, using specific kits (R&D Systems, Minneapolis, USA and eBioscience, San Diego, CA), according to the manufacturer’s protocol. The lowest detection limit of the assay was 156.25 pg/mL for sOSMR and 78.125 pg/mL for sgp130. Serum levels of OSM were not detected by the aforementioned methodology. The lowest detection limit of the assay was 15.2 pg/mL. Therefore, serum OSM levels were measured by western blot.

### OSM measurement

Protein expression of OSM in the serum of patients was performed by western blot.^21^An aliquot of serum from each patient was diluted 1:10 in MiliQ water, and the protein quantification was determined by the BCA Protein Assay kit according to the manufacturer’s instructions (Sigma-Aldrich®). After absorbance analysis, 50μg of proteins were electrophoresed on 10% polyacrylamide gel containing SDS (SDS-PAGE) and transferred to nitrocellulose membrane (GE Healthcare Life Sciences). Blocking of nonspecific sites was performed by incubating the membrane with TBST-BSA 5% at 4°C overnight. Membranes were incubated with the Rabbit Oncostatin M (OSM) (ColorBurst®) monoclonal antibody primary diluted 1:1000 in TBST-BSA 5% at 4 hours TA. After, membranes were incubated with respective HRP-conjugated secondary antibodies (1:5000). HRP-conjugated immunolabelled proteins were detected by the enhanced chemiluminescence method (ECL, GE Healthcare Life Sciences).

### Statistical Analysis

Data were analyzed using GraphPad Prism (version 6.0, San Diego, CA). The normality of the samples was verified with the D’Agostino test, and continuous variables were expressed as mean ± standard deviation ( *SD* ) if normally distributed or as medians [Interquartile Range [IQR] (25th–75th percentile)] if not showing Gaussian distribution. The median was also the criterion used to categorize the groups into higher and lower serum levels of sOSMR and sgp130. The nonparametric tested Kruskal-Wallis, followed by Brown-Forsythe post hoc test, along with the Spearman’s correlation for continuous variables, were used for the analysis of cytokines; the chi-square test and Fisher’s exact for categorical variables. The categorical variables were described through absolute and relative frequencies.

Furthermore, one-way analysis of variance (ANOVA), followed by Brown-Forsythe and Bartlett’s post hoc tests were used to evaluate OSM protein expression by Western Blot. Multivariate analysis was also performed to discern which parameters had independent predictive values in relation to serum levels of sOSMR, inferentially analyzed using Pearson’s chi-square or Fisher’s exact test. The regression analysis model used was logistic. P-values < 0.05 were considered to be statistically significant.

## Results

### Serum levels of sOSMR and sgp130

The clinical manifestations of the participants are summarized in Table 1 . Patients with CCS and ACS had significantly lower serum levels of sOSMR when compared to the control group, as shown in Figure 1a . Additionally, lower serum expression of sgp130 was detected in CCS and ACS when compared to the control, as shown in Figure 1b . No significant difference was detected in sOSMR and sgp130 serum levels between patients with CCS *versus* ACS.

**Figure 1:** – General distribution of the serum levels of sOSMR and sgp130 of the study population. A) Serum levels of sOSMR (soluble oncostatin M receptor) in pg / mL in the control subjects (CT) and the groups chronic coronary syndrome (CCS) and acute coronary syndrome (ACS). B) Serum levels of sgp130 (soluble glycoprotein 130) in pg / mL in the CT and the groups chronic coronary syndrome (CCS) and acute coronary syndrome (ACS). ****p <0.0001 vs control: Significant after analysis Kruskal-Wallis test.

### Serum levels of sOSMR according to sex and age

Figure 2a reveals that male patients with ACS and CCS had lower serum levels of sOSMR than women with the same conditions. Further, higher serum levels of sOSMR were detected in women with ACS when compared to CCS.

**Figure 2:** – General distribution of the serum levels of sOSMR by gender and age. A) Control subjects (CT) and the groups chronic coronary syndrome (CCS) and acute coronary syndrome (ACS) distribution between men and women serum levels of pg / mL (soluble oncostatin M receptor) sOSMR. B) Correlation between the levels of sOSMR (soluble oncostatin M receptor) in pg / mL and the age of ACS patients. ****p <0.0001 vs control: Significant after analysis Kruskal-Wallis test; * p <0.05 after Spearman’s correlation.

Among patients with ACS, age displayed a significant positive correlation with serum levels of sOSMR, as shown in Figure 2b . Correlation analysis was also performed on the CCS and control groups, but there were no significant results for either group [data not shown].

### Serum levels of OSM by western blot

The serum of 10 patients in each group was selected according to the criteria of sample representativeness (mean age of 60.5, 65.2, and 63.9 years in the CT, ACS, and CCS groups, respectively). Figure 3 indicates enhanced expression of OSM in ACS and CCS groups as compared to the control group. There was no significant difference in serum OSM levels between the ACS and CCS groups. Normalization for constitutional protein was performed with albumin, stained by the 0.01% Ponceau S technique,^22^since the sample used for the Western blot was whole blood.

**Figure 3:** – Protein expression of oncostatin M (OSM) among control subjects (CT), patients with acute coronary syndrome (ACS), and patients with chronic coronary syndrome (CCS) after normalization with albumin stained by Ponceau S. **** p <0.0001 vs control: Significant after analysis of variance (ANOVA).

### Serum levels and clinical variables

The results indicate a significant association between serum levels of sOSMR and patients with hypertension, dyslipidemia, stroke, revascularization, acute myocardial infarction (AMI), and smoker patients ( Table 1 of Supplementary Material ).

Looking at the side of medications, the use of statins, antiplatelet agents, insulin, blockers of calcium channels (BCCs), and antidiabetic drugs is positively associated with serum levels of sOSMR ( Table 2 of Supplementary Material ).

**Table 2:** – Logistic regression results for the percentage of patients with low levels of sOSMR according to independent variables with p <0.20 to be included in the analysis and p <0.20 to remain in the model

Variable	Bivariate		Adjusted	
	
	OR and 95.0% CI	p-value	OR and 95.0% CI	p-value
**Gender**		0.026*		
Men	2.05 (1.09 a 3.89)		1.82 (0.89 a 3.75)	0.102
Women	1.00		1.00	
**Age**		0.091		
Up to 60 years	1.68 (0.92 a 3.09)		2.28 (1.12 a 4.65)	0.023*
Over 60 years	1.00		1.00	
**Hypertension**		0.041*		
Yes	2.19 (1.02 a 4.64)		2.69 (1.15 a 6.30)	0.023*
2No	1.00		1.00	
**Dyslipidemia**		0.013*		
Yes	1.00		1.00	
No	2.32 (1.86 a 4.52)		2.56 (1.19 a 5.51)	0.016*
**Acute Myocardial Infarction**		0.001*		
Yes	1.00		1.00	
No	3.01 (1.54 a 5.90)		2.99 (1.41 a 6.32)	0.004*
**Antiplatelet agent**		0.005*		
Yes	1.00		1.00	
No	2.46 (1.31 a 4.62)		2.56 (1.25 a 5.25)	0.010*
**Blocker of calcium channels**		0.028*		
Yes	1.00		1.00	
No	3.15 (1.08 a 9.19)		4.13 (1.26 a 13.54)	0.019*

* significance values (p-values <0.05).

Table 2 shows that circulating serum levels of sOSMR were independently associated with sex, age, hypertension, lack of history of dyslipidemia and acute myocardial infarction, and non-use of antiplatelet agents, and BBCs.

## Discussion

The present study demonstrated not only diminished serum expression of sOSMR but also enhanced concentrations of serum OSM in patients with either CCS or ACS.

OSM appears in the heart following cardiac damage in order to promote cell survival and tissue repair.^23^In Hu et al.’s (2017) study, OSM attenuated the remodeling of the left ventricle and restored the density of mitochondrial ridges.^6^A fragmented soluble form of the OSM receptor was identified (i.e. sOSMR), which was responsible for an antagonist activity in the OSM receptor.^16 , 17^

Furthermore, we detected serum expression of OSM in CAD patients via western blot analysis. The absence of detection via ELISA might appear similar in difference in epitope recognition of the antibody used in this study or OSM serum levels may be below the detection limit of the ELISA. Of note, several other factors, including the presence of soluble receptors and antagonistic receptors, are also known to influence the quantification of cytokines in serum.^24^

On one hand, the literature suggests that OSM is a rare cytokine (i.e., not a commonly expressed cytokine) in which its elevated levels in the serum indicate tissue protection and repair after heart diseases.^23^One the other hand, Ikeda et al.’s (2021) study suggested a positive association between OSM serum levels and the development of stenosis.

The binding of OSM in the sOSMR requires the activation of gp130 and results in the inhibition of OSM activity, signaling involved in tissue repair after a heart injury.^11^In our study, elevated levels of OSM were observed in patients with cardiac injury when compared to controls, whereas decreased levels of sOSMR and sGP130 were observed in the same group of patients. The control group showed no heart injury or tissue damage that would endogenously raise mechanisms of repair, such as the enhancement of OSM expression as observed in the ACS and CCS groups. Thus, our results suggest that the increased expression of OSM in patients with ACS and CCS may indicate an important role in the pathophysiological mechanism of cardiac injury. However, further studies on the follow-up of disease evolution and serum expression of OSM/sOSMR/sGP130 should be performed to clarify the hypothesis of tissue damage or repair.

In spite of the data that OSM might promote OR expression,^12^few attempts have been made to explore such a signaling.^25^One hypothesis leads the way to the binding of sOSMR in OSM (i.e., OSMRβ), which might interrupt OSM activation. Blocking OSM signaling requires the action of sgp130. In fact, in the present study, we observed that CAD patients also have low levels of sgp130 when compared to the control group.

However, in the literature the association between sgp130 levels and cardiovascular diseases is controversial. A study of an elderly population with heart failure has shown that increased levels of sGP130 are related to cardiovascular mortality.^26^In addition, high sGP130 levels predict poor prognosis in patients with a history of myocardial infarction.^27^On the other hand, case-control research based on a much larger population has proposed that high sGP130 levels have protective effects against the occurrence of myocardial infarction.^28^A serological study has indicated that both patients with CAD in an unstable condition have significantly declined levels of endogenous sgp130. Furthermore, a recent study has demonstrated that sgp130 levels were significantly lower in patients with unstable or progressive CAD.^29^Zhou et al.’s (2020) study also found that the sGP130 level of 136.01 ng/mL was an effective cut-off point to predict CAD.^30^Likewise, we also suggested that sgp130 levels could be useful biomarkers for CAD identification. A positive correlation was observed between serum levels of OSM and the presence of patients with CAD, corroborating Ikeda et al.’s (2021) study.

High levels of OSM among patients with CAD may raise compensatory mechanisms for cell survival.^31^In Wahl et al.’s (2001) study, OSM balances inflammatory responses, suppressing inflammation, in murine models of chronic inflammatory diseases, including rheumatoid arthritis and multiple sclerosis.^32^Our results also indicate that age correlates with serum levels of sOSMR. Hartel et al.’s (2005) study showed otherwise. They observed that the levels of cytokines, such as TNF, INF-γ, and IL-2, increase progressively with age.^33^Our studies suggested that the lower the expression of OSM in elderly patients, the less the probability of CAD development; therefore, OSM might enhance protection in such a population. On the contrary, the literature has revealed that the severity of CAD increases with age, which has been attributed to the higher prevalence of the physical obstruction of the coronary arteries caused by atherosclerosis.^34 , 35^

Regarding the sex of patients, the results suggest that males have lower serum levels of sOSMR than females. Past studies have shown that the presence of estrogen during the fertile period prolongs the onset of atherosclerotic disease in females.^36 , 37^A clinical study by Women’s Ischemia Syndrome Evaluation in 2003 revealed that young women with endogenous estrogen deficiency have a sevenfold greater risk of developing atherosclerosis.^38^In the absence of estrogen’s cardioprotective benefit, men presumably require the activation of the OSM pathway in compensation.

Our study presented some limitations, including a small number of samples, and the heterogeneity of the type of medication used by the participants. Furthermore, it would be interesting to associate sOSMR and sgp130 with classical markers of tissue damage and lipid metabolism involved in coronary angiography. In summary, a Central Illustration to clarify ideas, is presented below with the main results of this article.

**Central Illustration:** : Decreased Serum Levels of Soluble Oncostatin M Receptor (sOSMR) and Glycoprotein 130 (sgp130) in Patients with Coronary Artery Disease

Central Figure summarizing the main results of this research.

## Conclusions

Our data suggest that increased serum levels of OSM and decreased levels of sOSMR and sGP130 in patients with cardiac injury may play an important role in the pathophysiological mechanism of the disease. Furthermore, lower levels of sOSMR were associated with gender, age, hypertension, and the use of medications. Additional studies about the follow-up of disease outcome and OSM/sOSMR/sGP130 serum expression with an enhanced number of patients, controlled therapy, and association with biomarkers of tissue damage or repair must be conducted to strengthen our hypothesis.

## * Supplemental Materials

For additional information, please click here .
